# Individual trajectories for recovery of neocortical activity in disorders of consciousness

**DOI:** 10.1371/journal.pcbi.1013659

**Published:** 2025-11-11

**Authors:** Prejaas K. B. Tewarie, Romesh Abeysuriya, Rajanikant Panda, Pablo Nùñez, Marie M. Vitello, Glenn van der Lande, Olivia Gosseries, Aurore Thibaut, Steven Laureys, Gustavo Deco, Jitka Annen

**Affiliations:** 1 Joint International Research Unit on Neuroplasticity, CERVO Brain Research Center, University of Laval, Quebec, Canada; 2 Sir Peter Mansfield Imaging Centre, School of Physics, University of Nottingham, Nottingham, United Kingdom; 3 Computational Neuroscience Group, Center for Brain and Cognition, Universitat Pompeu Fabra, Barcelona, Spain; 4 Disease Elimination Program, Burnet Institute, Melbourne, Australia; 5 Department of Epidemiology and Preventive Medicine, Monash University, Melbourne, Australia; 6 Coma Science Group, GIGA-Consciousness, University of Liege, Liege, Belgium; 7 Centre du Cerveau, University Hospital of Liege, Liege, Belgium; 8 Department of Neurology, University of California San Francisco, San Francisco, California, United States of America; 9 School of Psychological Sciences, Monash University, Melbourne, Australia; 10 Department of Neuropsychology, Max Planck Institute for Human Cognitive and Brain Sciences, Leipzig, Germany; Newcastle University, UNITED KINGDOM OF GREAT BRITAIN AND NORTHERN IRELAND

## Abstract

The evolution from disturbed brain activity to physiological brain rhythms can precede recovery in patients with disorders of consciousness (DoC). Accordingly, intriguing questions arise: What are the pathophysiological factors associated to disrupted brain rhythms in patients with DoC, and are there potential pathways for individual patients with DoC to return to normal brain rhythms? We addressed these questions at the individual subject level using biophysical simulations based on electroencephalography (EEG). The main findings are that unconscious patients exhibit a loss of excitatory corticothalamic synaptic strength. Synaptic plasticity in this excitatory corticothalamic circuitry facilitates the return of physiological brain rhythms, characterized by the reappearance of spectral peaks and flattening of the aperiodic (1/f) component of the power spectrum, in the selection of patients with DoC, particularly in those who are minimally conscious. The extent to which this occurred was correlated with cerebral glucose uptake. The current findings emphasize the importance of excitatory thalamocortical activity in reestablishing normal brain rhythms after brain injury and show that biophysical modelling of the corticothalamic circuitry could help select patients who might be potentially receptive to treatment and undergo plasticity.

## Author summary

Following acute brain injury, some patients remain in a disorder of consciousness (DoC), ranging from unresponsive wakefulness to minimal awareness. Clinicians track recovery using electroencephalography (EEG), a technique that measures the electrical rhythms of the brain. Normal brains exhibit typical alpha and theta rhythms; in DoC, these rhythms are less apparent. We posed two challenging questions: which features of the brain’s wiring are most disrupted in DoC, and by what biological pathways can normal rhythms recover? Using a biophysical model fitted to each patient’s EEG, we found that the primary deficit lies in weakened excitatory connections from the thalamus to the cortex. This pathway helps organize widespread cortical activity. When we allowed these thalamo-cortical connections to strengthen in the model (a form of synaptic plasticity), physiological rhythms reappeared in a subset of subjects, but not when cortical connections were strengthened in isolation. The extent of modelled recovery was correlated with cerebral glucose metabolism as measured by PET, implying an association between maintained brain energy and the capacity for plasticity. Our findings show that simulating personalized thalamo-cortical dynamics from common EEG would allow identification of patients with most favorable response to recovery-promoting treatments.

## Introduction

Disorders of consciousness (DoC) occur in a small proportion of comatose survivors with severe brain injury. While the etiology varies among patients, common etiologies include severe traumatic brain injury (TBI), stroke, and cardiac arrest. Irrespective of the etiology, these patients can be grouped into the unresponsive wakefulness syndrome (UWS), characterized by the presence of eye-opening and reflexive behaviors, and the minimally conscious state (MCS), characterized by inconsistent conscious behaviors, such as command following or visual pursuit. There is widespread reduction of cerebral metabolism, commonly measured using [^18^F]Fluorodeoxyglucose Positron Emission Tomography (FDG-PET), and disrupted neocortical activity in DoC due to damaged neuronal circuitry at the cellular level [1]. Restoration of cerebral activity (e.g., measured with electroencephalography (EEG) or FDG-PET) is believed to precede clinical recovery [2,3]. Patients in whom preservation of cerebral activity was observed in the absence of behavior were referred to as MCS stars (MCS*) [4]. Hence, exploring potential routes for the restoration of cerebral activity is crucial for understanding clinical recovery in patients with DoC.

Cortical activity is commonly measured by EEG. This activity represents the electrical signals generated by neural populations under the skull, orchestrated by corticocortical and thalamocortical connections. EEG signals in healthy subjects can usually be decomposed into an aperiodic component ($1/{f\;}^{k}$ component) and a periodic component or spectral peaks [5]. A proposed qualitative model for the evolution of the EEG power spectrum in DoC is often referred to as the “ABCD” model, derived from the mesocircuit hypothesis [6]. This model states that cortical activity in severe brain damage leading to UWS is restricted to the aperiodic part, which is dominated by a high delta power (i.e., < 4 Hz). Recovery of consciousness is believed to co-occur with the emergence of a spectral peak in the lower frequency range (around 7 Hz), with a shift towards higher frequencies (first around 10 Hz and potentially around 20 Hz) during further recovery, and flattening of the aperiodic $1/{f\;}^{k}$ slope [6]. The relevance of EEG measurements in DoC is underscored by the notion that the re-emergence of physiological EEG features is associated with behavioral recovery [2,7–9] and glucose uptake [10].

Several hypotheses may explain the recovery of the EEG power spectrum in patients with DoC. One of these hypotheses, the meso-circuit hypothesis, states that the return of spectral peaks and flattening of the aperiodic part in the EEG power spectrum is a result of the restoration of excitation in the thalamocortical circuit and, more specifically, a more widespread cortico-striato-pallido-thalamic network [11]. This hypothesis also postulates widespread deactivation of excitatory synaptic activity across the cerebral cortex, resulting in a global hyperpolarized state in DoC, which is known as “disfacilitation” [6]. Recovery in this situation would require boosting the excitation to induce a shift from this hyperpolarized state. Restoration of excitatory processes presumably depends on neuronal repair and synaptic plasticity [8], and both are assumed to occur in DoC to some extent [12]. The potential roles of neuroprotective and neurostimulating drugs that induce cerebral plasticity, resulting in partial recovery of consciousness, underscore this view [13].

The mechanisms underlying the recovery of EEG power spectra in patients with DoC cannot be directly inferred from the EEG data. However, using biophysical models of macroscopic brain activity, we cannot only infer biophysical model parameters (e.g., synaptic properties not measured empirically with EEG) from EEG power spectra of individual patients but also model subject-specific synaptic plasticity [14]. Our biophysical model includes excitatory and inhibitory intracortical, intrathalamic, and corticothalamic synaptic strengths as well as synaptic time constants and axonal conduction delays. This model allowed us to test the roles of excitation and inhibition in thalamocortical synaptic connections. It allowed us to identify the circuits that are most affected in patients with DoC, for example, thalamocortical or intracortical circuits. This allowed us to model the subject-specific plasticity of synapses and their effects on brain rhythms. Well-known types of plasticity commonly used in corticothalamic biophysical models are Hebbian and homeostatic plasticities [15–17]. The former is a positive feedback-mediated form of plasticity in which synapses between presynaptic and postsynaptic neurons that are coincidently active are strengthened. The latter is a negative feedback-mediated form of plasticity, also known as synaptic scaling, which maintains network activity at the initial level.

In the current work, we will address three questions: 1) What type of cortical or corticothalamic synaptic damage in our biophysical model is most consistent with disrupted EEG in patients with DoC? 2) What are the potential cortical or corticothalamic routes of plasticity that lead to neurophysiological recovery in individual patients with DoC? 3) To what extent do the routes for neurophysiological recovery relate to metabolic preservation as potential biomarkers of plasticity?

## Results

### Estimating corticothalamic model parameters for disturbed EEG patterns in patients with DoC

**Fig 1:**
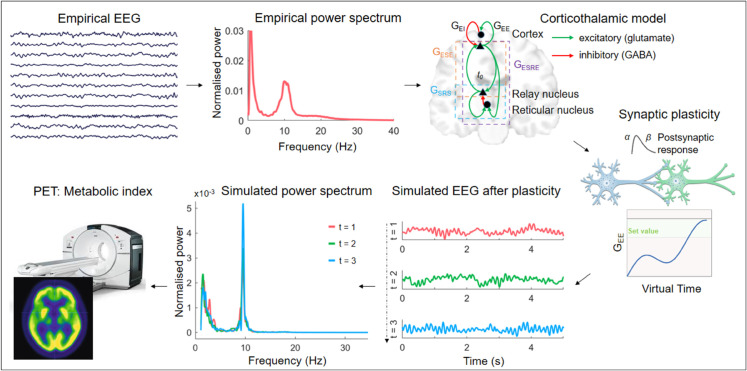
Overview of the pipeline. The first part of the analysis entails the estimation of biophysical model parameters from individual EEG data. We use a Markov Chain random walk to estimate biophysical parameters from subject-specific whole-brain power spectra. This is followed by subject-specific simulations using either corticothalamic or cortical plasticity. Potential links with available FDG-PET data are further analysed. Abbreviations: excitatory corticothalamic synaptic strengths (*G*_*ESE*_), inhibitory corticothalamic synaptic strengths (*G*_*ESRE*_), Excitatory cortical synaptic strengths (*G*_*EE*_), inhibitory cortical synaptic strengths (*G*_*EI*_), intrathalamic synaptic strengths (*G*_*SRS*_), synaptic decay and rise constants (*α* and *β*), corticothalamic time delay (*t*_0_), electroencephalography (EEG).

We analyzed EEG data from 145 DoC patients (MCS (n=95), MCS* (n=12), and UWS (n=38)) and 30 healthy control subjects. The power spectra for each subject were estimated and averaged across (occipital, temporal and parietal) electrodes, resulting in a single power spectrum per subject. We used a Markov chain random walk to estimate nine parameters of the biophysical corticothalamic model [14]. This corticothalamic model includes a cortical excitatory and cortical inhibitory population, a thalamic relay, and a reticular population (Fig 1). The firing activity of the presynaptic populations modulates the postsynaptic membrane potential of a population. The impact of presynaptic input on the postsynaptic membrane potential also depends on the mean number of synapses between the presynaptic b and the postsynaptic population a (modelled as synaptic strength $\nu_{ab}$) and on the closing and opening rates of synaptic channels characterized by synaptic decay and rise constants (*α* and *β*). The average postsynaptic membrane potential is transformed into firing activity in the cell bodies of a population. This process results in the propagation of activity in a closed loop between the thalamic and cortical populations, where the propagated firing rate between the thalamus and cortex is delayed by *t*_0_. The synaptic parameters $\nu_{ab}$ are transformed in a linear version of the model to the gain parameters *G*_*ab*_ and closed-loop parameters $G_{aba}=G_{ab}G_{ba}$. Given this transformation, the gain parameters did not strictly indicate the equivalence of synaptic strengths. Note that the model’s proxy for EEG is a derived measure of cortical excitatory activity, appearing in low-pass filtered form as a result of volume conduction through the skull, cerebrospinal fluid, and scalp.

**Fig 2:**
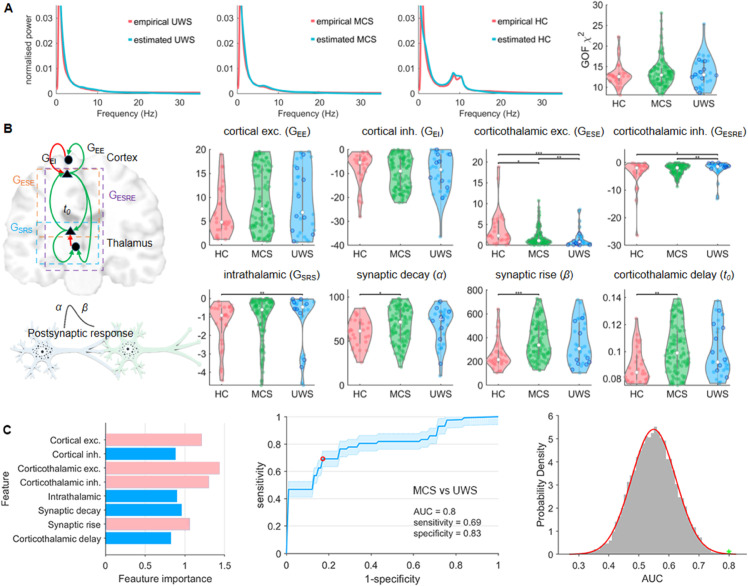
Estimating corticothalamic model parameters in DoC. Panel A shows the group-averaged empirical power spectra and model-estimated power spectra for the different groups alongside the goodness of fit of the estimation for the different groups. Panel B presents a graphical representation of the model parameters and their estimates across the different groups. Blue circles in the UWS group denote MCS* patients. Both excitatory and inhibitory corticothalamic synaptic strengths differ between MCS and UWS. Panel C illustrates the results of our classification analysis of MCS vs. UWS, showing the feature importance for each parameter (left), the receiver operating characteristic (ROC) curve (middle), and the surrogate distribution of AUC values (right). The genuine AUC value is depicted in green. The features that remained in the final model are depicted in pink (left panel), with corticothalamic excitatory *G*_*ESE*_ being the feature or parameter that contributes most strongly to the classification. Abbreviations: healthy controls (HC), minimal conscious state (MCS), unresponsive wakefulness syndrome (UWS), excitatory corticothalamic synaptic strengths (*G*_*ESE*_), inhibitory corticothalamic synaptic strengths (*G*_*ESRE*_), Excitatory cortical synaptic strengths (*G*_*EE*_), inhibitory cortical synaptic strengths (*G*_*EI*_), intrathalamic synaptic strengths (*G*_*SRS*_), synaptic decay and rise constants (*α* and *β*), corticothalamic time delay (*t*_0_). *=p<0.05,**=p<0.01,***=p<0.001. (FDR corrected).

Fig 2A shows the empirical power spectra (red) and model-estimated power spectra (blue) averaged across the subjects within each group. Power spectra from patients with UWS are mostly characterized by an aperiodic component without a spectral peak, whereas power spectra from patients with MCS are predominantly characterized by an aperiodic component with a subtle peak in the theta-band (4-8 Hz). The fraction of patients with a spectral peak in the (lower) frequency bands was 0.21 for UWS and 0.44 for MCS (p = 0.026, χ = 4.97). The power spectrum of healthy control subjects was characterized by an aperiodic component (1/f part) and a spectral peak in the alpha band at approximately 10 Hz. For all groups, the model-estimated power spectra (blue curves) provide a close approximation to the empirical power spectra, as evident from Fig 2A, both visually and in terms of the goodness-of-fit (GOF) in the rightmost panel. There was no significant difference in GOF between the groups (p > 0.05). Figs A–C in S1 File show the estimated and empirical power spectra for the individual patients. This indicates that the individual fits only captured the global shape of the power spectra, including the most prominent peaks. This may lead to the omission of details in the estimated spectra, but avoids overfitting.

The groups did not differ in cortical excitatory (*G*_*ee*_) or cortical inhibitory (*G*_*ei*_) synaptic gains (Fig 2B). However, all groups differed in terms of excitatory corticothalamic synaptic gains (*G*_*ese*_) and inhibitory corticothalamic synaptic gains (*G*_*esre*_). In particular, the excitatory corticothalamic synaptic gain (*G*_*ese*_) could differentiate between the MCS and UWS groups. Patients in the UWS group were characterized by areduced excitation and increased inhibition of the thalamocortical system. There was a loss of inhibition in the intrathalamic loop in DoC (*G*_*srs*_); however, this could not differentiate between UWS and MCS. Furthermore, there were considerably longer synaptic decay and rise constants (*α* and *β*) for the MCS group compared to the healthy control (HC) group, although the UWS group did not differ from either the HC or MCS group. The same held true for the corticothalamic time delay *t*_0_ with longer time delays in the MCS group than in the healthy control group and no differences between the MCS and UWS groups were observed. Of note, the aforementioned significant differences between the groups could not be explained merely by etiology (see Fig D in S1 File).

The univariate analysis was extended with a multivariate approach to classify MCS or differentiate it from UWS using multiple parameters. Initially, all parameters were included, resulting in an AUC of 0.77. After feature selection with a sequential leave-one-out method, a model with four key parameters emerged (see pink bars in Fig 2C left panel). Among these, corticothalamic parameters contributed most significantly to classification, particularly excitatory synaptic strength. Using corticothalamic excitatory and inhibitory strengths, cortical excitatory synapses, and synaptic rise time, this refined model achieved an AUC of 0.8 (Fig 2C middle panel). Statistical comparison with surrogate data confirmed that the obtained AUC value could not be obtained by chance (Fig 2C right panel).

### Potential routes for recovery of EEG activity in individual patients with DoC

We studied two potential mechanisms for recovery of EEG activity in patients with DoC: 1) the role of excitatory synaptic plasticity in the cortex and 2) the role of excitatory synaptic plasticity in the corticothalamic loop. We employed the full non-linear model in this context and simulated model Eqs 1–3 (see Materials and Methods) with subject-specific model parameters obtained from the previous analysis (Fig 2B). The synaptic parameters *G*_*ab*_ translate to $\nu_{ab}$ in the fully nonlinear model regime. We applied synaptic plasticity (Eq 14) to the excitatory corticothalamic loop $\nu_{es}$ and the excitatory intracortical loop $\nu_{ee}$. Hence, in our simulations, synaptic plasticity unfolds on a slow virtual timescale relative to the rapid corticothalamic dynamics. We define ‘evolution of synaptic plasticity’ as gradual changes in synaptic parameters that manifest as systematic alterations in the power spectrum over simulated virtual time.

**Fig 3:**
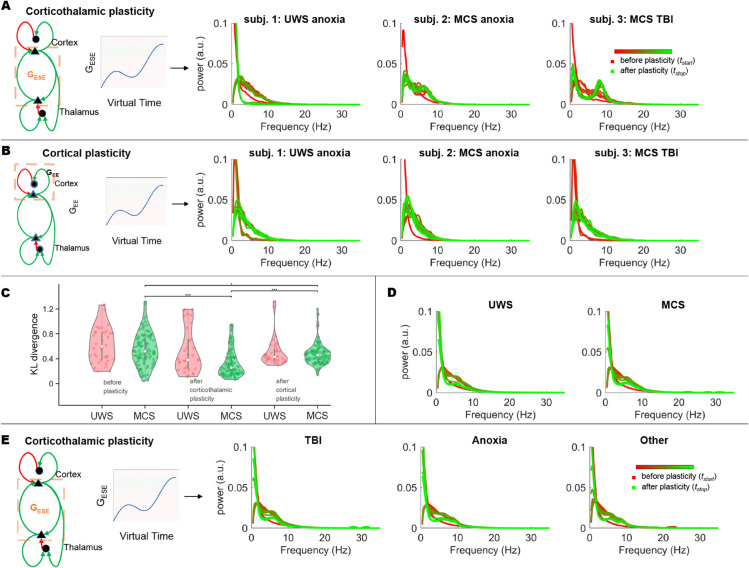
Modelling synaptic plasticity in DoC. Panels A and B show simulated power spectra for individual subjects during excitatory corticothalamic (A) and excitatory cortical synaptic plasticity (B). Panel C shows the Kullback-Leibler (abbreviated to KL) divergence during either cortical plasticity (D) or corticothalamic plasticity (E). Simulated power spectra were assessed for their similarity (low KL value) with those from healthy control subjects, with significant differences denoted by ** for *p* < 0.001 and* for *p* < 0.05. Panel D shows the group-averaged power spectra during the course of corticothalamic plasticity for the UWS and MCS groups. The same group-averaged power spectra are illustrated for different etiologies (E). Abbreviations: healthy controls (HC), minimal conscious state (MCS), unresponsive wakefulness syndrome (UWS), excitatory corticothalamic synaptic strengths (*G*_*ESE*_), inhibitory corticothalamic synaptic strengths (*G*_*ESRE*_), Excitatory cortical synaptic strengths (*G*_*EE*_), inhibitory cortical synaptic strengths (*G*_*EI*_), intrathalamic synaptic strengths (*G*_*SRS*_), synaptic decay and rise constants (*α* and *β*), corticothalamic time delay (*t*_0_).

Fig 3A shows examples of the subject-specific simulations of corticothalamic plasticity. The red line shows the empirical power spectrum superimposed on several power spectra during the evolution of synaptic plasticity (transformation from red to green over time). The effects of corticothalamic synaptic plasticity differed between the subjects. While some subjects developed a spectral peak in the theta band or even the alpha band, other subjects did not develop a clear spectral peak despite the induction of corticothalamic synaptic plasticity. For example, a patient with UWS due to anoxia did not show an apparent spectral peak after corticothalamic plasticity, whereas a patient with MCS due to TBI developed an evident alpha peak only after the corticothalamic plasticity was set (Fig 3A). The implementation of cortical plasticity alone produced a different picture (Fig 3B). Although cortical synaptic plasticity led to a different slope of the power spectrum, no spectral peak was induced by cortical plasticity alone in any of the patients. Fig 3B shows the power spectra for the same groups demonstrated in Fig 3A.

The results of the individual spectra suggest that spectral peaks are more likely to occur during the progression of synaptic corticothalamic plasticity in patients with MCS than in those with UWS. The fraction of patients who showed a clear peak after corticothalamic plasticity that was not present in the initial and original empirical data was 0.2 for UWS and 0.45 for MCS (p = 0.019, χ = 5.43). As neurophysiological recovery involves restoring physiological rhythms similar to those observed in healthy individuals, we assessed recovery by measuring the difference between the power spectra of subjects with DoC and healthy controls, quantified using the Kullback-Leibler divergence, where a smaller divergence indicates a greater recovery. Fig 3C shows that for patients with MCS, both corticothalamic and cortical plasticity resulted in a power spectrum more similar to that of healthy controls. At the same time, this was much more pronounced for corticothalamic plasticity than for cortical plasticity. This effect was probably driven by a significantly stronger occurrence of a spectral peak in the higher levels of consciousness than in the lower levels of consciousness (Fig 3D). Finally, we analyzed the effect of corticothalamic plasticity in patients with different etiologies, as the contributions of certain etiologies may differ between the MCS and UWS groups (Fig 3F). The group-averaged power spectra showed that the reoccurrence of a spectral peak may be more prominent in the TBI subgroup than in the anoxic subgroup (Fig 3F).

### Neurophysiological correlates of FDG-PET findings in DoC

**Fig 4:**
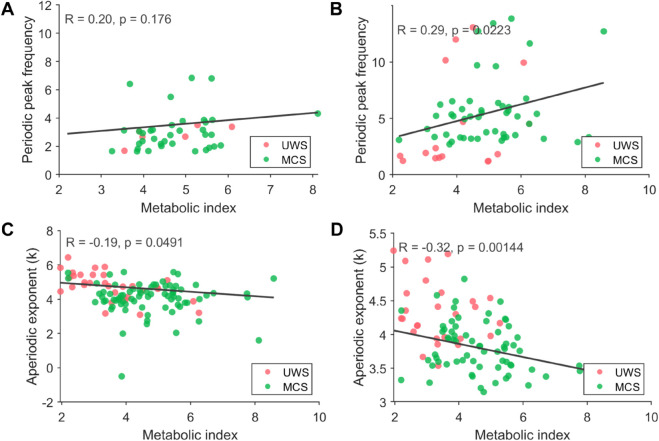
The relationship between modelled synaptic plasticity and FDG-PET findings in DoC. Panel A shows scatter plots and correlations between the peak frequencies of empirical EEG data with the metabolic index from FDG-PET. Panel B shows the same for simulated data. Panels C and D show scatter plots, and correlations between the aperiodic exponent and metabolic index. Significant correlations were especially observed for the modelled data. Note that the x-coordinates of panel A and B differ because fewer patients exhibited identifiable spectral peaks (particularly in UWS) in the empirical data, resulting in less peak-frequency data than aperiodic data.

To better characterize the relationship between cerebral integrity and the ability to recover physiological rhythms, we associated spectral features, such as peak frequency and the aperiodic exponent, with metabolic activity. We extracted the peak frequency of the empirical power spectrum for all participants who had a peak present before the application of plasticity (UWS, n = 5 of 36; MCS, n = 42 of 95). This was also performed for the power spectra extracted from the simulated EEG data after corticothalamic plasticity (UWS, n = 13 of 36; MCS, n = 80 of 95). The scatter plots in Fig 4A show the relationship (or lack thereof) between the metabolic index of the best-preserved hemisphere and peak frequency from the empirical power spectra. However, no correlation was observed between these two entities in the across both groups (Fig 4A). However, for the modelled power spectra obtained after corticothalamic plasticity, we observed moderate correlations with the metabolic index across groups (Fig 4B).

To further explore the association between cerebral integrity and the recovery of physiological rhythms, we examined the relationship between the aperiodic exponent of the power spectrum and metabolic activity. The aperiodic exponent, a measure often associated with underlying neural excitability, was extracted from both empirical and simulated EEG data. For empirical data, the UWS group had significantly higher aperiodic exponents than the MCS group (*p* = 0.009, *Z* = 2.6). This finding is in agreement with the mesocircuit hypothesis, which suggests that a flattening slope is associated with better clinical status. For the empirical power spectra, scatter plots in Fig 4B illustrate a weak negative relationship between the aperiodic exponent from empirical EEG data and the metabolic index in both the UWS and MCS groups. In the simulated EEG data after corticothalamic plasticity, there was again a higher exponent in the UWS group compared to the MCS group (*p* = 0.001, *Z* = 3.2). This was again accompanied by a moderate negative correlation between the aperiodic exponent and the metabolic index across groups (Fig 4B). This association in the modelled data suggests indeed that flattening of the aperiodic exponent may capture aspects of neuronal excitability that align more closely with metabolic integrity post-plasticity.

## Discussion

Clinical recovery in patients with DoC may be dependent on sufficient cerebral metabolism and the recovery of physiological brain rhythms, as measured by EEG (6). Insight into the evolution of physiological EEG rhythms in DoC requires knowledge of the most important pathophysiological determinants of disturbed EEG activity. Using parameter estimation, we demonstrated that disturbed brain rhythms in DoC were likely the result of delayed propagation of neuronal activity between the thalamus and cortex, delayed synaptic responses, and loss of synaptic strength within the thalamus, especially loss of synaptic strength between the thalamus and cortex. Corticothalamic synaptic strength was not only lower in patients with DoC but also sensitive in differentiating between patients with MCS and UWS. The important role of corticothalamic synapses was strengthened by the observation that plasticity in these synapses specifically resulted in the recovery of physiological brain rhythms (e.g., theta and alpha rhythms) indicative of thalamocortical modulation in some patients. The extent to which this occurred correlated with whole-brain metabolic activity as measured by FDG-PET. Recovery of theta and alpha brain rhythms cannot be achieved merely by cortical synaptic plasticity.

The current findings are in agreement with the mesocircuit hypothesis, which states that the slow EEG activity observed in patients with DoC results from the quiescent activity of the central thalamus and complete deafferentiation of excitatory corticothalamic synapses (6). The mesocircuit model predicts that the power spectrum in DoC recovers its shape from a $1/{f\;}^{k}$ spectrum to a spectrum with a flattened aperiodic component superimposed by peaks in the theta and alpha bands. While theta activity may emerge only from cortical neurons, alpha activity may emerge from increased activity in the central thalamus and the restoration of corticothalamic synaptic connectivity [18]. However, to date, this prediction has mostly been verified indirectly using structural MRI [19], fMRI [20,21], FDG-PET [22], or in vivo animal studies [23]. The current findings reveal a more direct relationship between qualitative model predictions of the mesocircuit hypothesis and EEG findings in DoC. We damage to the excitatory corticothalamic synapses. The latter was also sensitive in distinguishing between MCS and UWS. Another observation in agreement with the mesocircuit hypothesis was that intrathalamic synaptic strength was weaker in patients with DoC than in healthy controls, which could lead to reduced excitatory output towards the cortex.

The presumed role of excitatory corticothalamic synapses in the recovery of EEG rhythms in DoC was further strengthened by the finding that the plasticity of corticothalamic synapses, rather than merely cortical synapses, could result in the re-emergence of the specral peaks in some patients. Interestingly, the extent to which the spectral peaks re-emerged was related to the metabolic index extracted from FDG-PET. The relationship between the modelled spectral peak and the metabolic index was stronger than that between the patients’ empirical spectral peak and their metabolic index. The relationship between the occurrence of spectral peaks on EEG after corticothalamic synaptic plasticity and the metabolic index suggests that the metabolic index from FDG-PET can encompass information about the potential to recover and decode the residual capacity of the thalamocortical system in the brain [2]. This suggests that the functional integrity of neurons, as measured using glucose, is a good index of potential local and global network interactions that give rise to higher peak frequencies. This finding is in agreement with a previous functional MRI study, which suggests that stronger connectivity (albeit within purely cortical networks) is related to a higher metabolic index in patients with DoC [24,25]. Furthermore, we observed that plasticity induced a spectral peak in a subset of patients. High response rates are biologically unrealistic in patients with severe brain damage, some of whom may never recover. In patients with DoC, recovery is generally less likely following anoxic brain injury - particularly when associated with global atrophy - than following local traumatic brain injury [26]. In line with these observations regarding spontaneous recovery, we found that patients with a traumatic brain injury had a higher probability of recovering a spectral peak than patients with anoxic brain injury. Finally, we observed that the proportion of patients in whom plasticity altered the power spectra aligns with treatment response rates reported in empirical studies reviewed by Thibaut et al. [27]. These important insights increase the biological plausibility and clinical relevance of this model.

This study has several potential clinical implications. As corticothalamic plasticity induces faster alpha oscillations in some patients, while failing to induce alpha oscillations in others, it could identify which subjects could be sensitive to (noninvasive) interventions in DoC [28], such as amantadine or transcranial direct current stimulation [27,29,30]. The results suggest that in some patients, overall synaptic and axonal damage is too severe, and promoting corticothalamic plasticity in these patients is insufficient to return to physiological brain rhythms. In some patients, there may be a residual capacity of the brain that can be assessed by promoting corticothalamic plasticity. For example, the results of deep brain stimulation of the central thalamus in patients with DoC have been mixed [31], potentially because of the suboptimal selection of patients with sufficient residual capacity, despite the observation that plasticity may be promoted by deep brain stimulation in other clinical populations [32]. Recent work has shown that low-frequency stimulation of the centromedian-parafascicular complex (CM-Pf) in the thalamus in DoC could promote the regeneration of whole-brain communication in the alpha band [33]. Hence, there may be a role for subject-specific corticothalamic modelling based on resting-state EEG data in the selection of patients for deep brain stimulation and potentially for other (non-invasive) interventions such as transcranial magnetic stimulation, transcranial direct-current stimulation, or vagal/median nerve stimulation [34,35]. Particularly for MCS* patients with higher recovery rates at the group level [2], the model would be valuable for predicting recovery. However, because the number of patients with MCS* in this study was relatively small, they were not included in a separate group. We denoted them with different colours in the plots, which showed that the potential plasticity in the UWS group was mainly driven by MCS* patients. Future studies using longitudinal data could assess whether this framework opens avenues for predicting therapeutic effects and spontaneous recovery in patients with DoC.

*Methodology and limitations.* fMRI model-based approaches to better characterize DoC have shown that low-level states of consciousness are characterized by segregated and less connected network states, potentially caused by increased inhibition [36–38]. These approaches are increasingly being studied; however, their direct clinical relevance is limited because of the complexity of data acquisition and analysis. An important methodological advancement of the proposed approach is plasticity induction in the modelled resting-state EEG data. This could be relevant not only to DoC but also to other pathologies. EEG-based modelling studies on DoC are scarce [39,40], although we envision their clinical applicability to be more direct. For example, the analysis and (plasticity-induced) peak detection can be fully automated. Some reflections on the model-based approach are warranted. Our results may be biased by the choice of biophysical model, as this model for healthy conditions is tuned to generate a 1/f spectrum superimposed by alpha oscillations from corticothalamic resonance due to a delay in propagation between the thalamus and the cortex [41]. However, previous work has demonstrated three possible ways to generate alpha oscillations in this specific model, including the corticothalamic circuitry and an intracortical circuit [42]. Further, we focused on the synaptic plasticity of excitatory synapses. However, in light of the mesocircuit hypothesis, the role of inhibition should also be studied, suggesting that disinhibition of the thalamus may play an important role in inducing excitatory input from the thalamus. More detailed biophysical models that incorporate the caudate and globus pallidus could be utilized to investigate the role of plasticity in disinhibition in future studies. Next, despite the existence of several other hypotheses on consciousness that could be applied in the context of DoC, such as integrated information theory [43] and the global neuronal workspace [44], these cannot be used to make quantitative predictions for EEG spectra from patients with DoC based on underlying neuronal circuits but require extraction of other features from EEG data [45]. Hence, we restricted our interpretation of our findings to the context of the mesocircuit hypothesis. An important limitation of this study is that we have focused on estimating parameters from the average power spectrum, rather than examining the spatial variability of power spectra and their corresponding parameters. However, this approach allowed us to significantly reduce the parameter space. Furthermore, our emphasis on global dynamics stems from the belief that (1) brain injury affects arousal and attention systems in general, with a pathophysiology independent from the nature of brain injury and (2), in light of recovery, especially global state changes rather than improvement in modular functions is most beneficial, as outlined in [39]. Future studies should investigate if model parameters and, eventually, plasticity vary significantly across regions, having potential implications for the recovery of (covert) awareness and function.

In conclusion, we demonstrated the advantage of biophysical modelling in individual subjects with DoC, showing that the recovery of the corticothalamic circuitry is accompanied by the reappearance of physiological brain rhythms in some patients with DoC. Although these findings underscore the predictive ability of the mesocircuit hypothesis, they may also have potential clinical implications. Examples include the prediction of recovery in patients with DoC and aid in the selection of patients with sufficient residual brain capacity for (non-invasive) treatment. Future work is needed to verify whether the recovery of the excitatory corticothalamic circuitry results in good neurological recovery.

## Methods and materials

### Ethics statement

This study was approved by the Ethics Committee of the University Hospital of Liège. All healthy participants and the legal surrogates of patients provided written informed consent to participate in the study.

### Experimental design

We included 145 patients (58 females, mean age 40 years ± 17 years) and 30 healthy control subjects (controls; 14 females, mean age 43 years± 15). Patients were diagnosed with MCS (n=95), MCS* (n=12), or UWS (n=38). The etiologies were traumatic brain injury in 76 patients, anoxia after cardiac arrest in 50 patients, mixed anoxia after cardiac arrest and traumatic brain injury in 7 patients, stroke/hemorrhage in 19 patients, and other etiologies, such as metabolic encephalopathy or infection of the central nervous system, in 3 patients. The average time between injury and hospitalization/scanning was 2.3 years, with a standard deviation of 3.5 years. The level of consciousness of the patients was assessed using the Coma Recovery Scale-Revised (CRS-R) [46], which was repeated at least five times to minimize clinical misdiagnoses [47]. The patients’ diagnoses were based on the best behavioral scores obtained over repeated CRS-R assessments during the week of hospitalization.

### EEG data acquisition and preprocessing

Part of the EEG data was used in previous studies [2,48–50]. Our preprocessing pipeline adhered to the recent guidelines and recommendations for EEG data analysis [51]. Briefly, EEG recordings of 20–25 min were obtained from patients with DoC and healthy controls. The data were acquired using a high-density EEG system with a sampling frequency of 250 Hz. Data from some patients were acquired at a sampling frequency of 500 Hz. Data from these patients were downsampled to 250 Hz. An Electrical Geodesic Inc. EEG system with 256 channels was employed, and a saline solution was used. During data collection, patients were kept awake as much as possible. An examiner was present during the acquisition to ensure this. EEG data were imported into MATLAB version R2021a for further analysis, partly using FieldTrip [52]. EEG data from the neck, forehead, and cheek channels were discarded because they can be easily contaminated by muscle artifacts, resulting in 150 channels that were used for further analysis. EEG data were segmented into epochs of two seconds, and epochs with signals that had an amplitude exceeding 100 *μ*V were rejected automatically. The remaining bad epochs were rejected via visual inspection (Jitka Annen). Data were referenced to a common average montage [2]. The EEG data were further preprocessed using a zero-phase sixth-order Butterworth bandpass filter of 0.5–40 Hz. After preprocessing, we computed the power spectrum for each channel and averaged it across the channels. Only the scalp channels covering the parietal, temporal, and occipital areas were included to compute the grand average across the channels. Other channels, such as those covering the frontal areas and jaws, were excluded. Power spectra were computed using Welch’s method with windows of 5 s with 2.5 s overlap.

### FDG-PET data

Patients fasted for at least six hours prior to the FDG-PET procedure. FDG-PET data were acquired after intravenous injection of 150–300 MBq of FDG on a Philips Gemini TF PET-CT scanner. The PET data were spatially normalized and smoothed using a Gaussian filter with a full-width at half-maximum value of 14 mm. The data were recorded in a single 12-minute emission frame after a 30-minute uptake phase, and the images were corrected for attenuation. Two FDG-PET analyses were performed. First, a statistical evaluation of relatively preserved and hypometabolic regions was performed, comparing them to those of healthy volunteers, as described previously [25]. The brain FDG-PET SUV of each patient with an unequivocal and reliable bedside diagnosis of UWS or MCS was visually inspected by three experts in the analysis of FDG-PET of DoC patients. The patients were blinded to their clinical diagnoses. Patients with clinical UWS were subsequently qualitatively classified as MCS* [4] based on the relative preservation of metabolism in the frontoparietal network [53]. Second, for quantitative assessment of glucose metabolism, the FDG-PET metabolic index of the best-preserved hemisphere (MIBH) was calculated to approximate the cerebral metabolic rate of glucose at the single-subject level [54]. As previously described [53], individual images were registered on a population-specific FDG-PET template. Images were segmented into the left and right cortices, as well as extracerebral tissue. Cortical uptake was normalized based on the metabolism of extracerebral tissue in healthy volunteers and scaled to a value between 0 and 1, based on the mean activity of the extracerebral tissue. Finally, the MIBH was calculated as having the highest mean metabolism in both hemispheres.

### Corticothalamic mean-field model

We employed a corticothalamic mean-field model [41], which describes the aggregate activity of a neuronal population in terms of their firing activity $\phi_{a}$ and mean membrane potential $V_{a}$ with a being either *e,i,r*, or *s*. The corticothalamic mean-field model encompasses two cortical populations (excitatory (*e*) and inhibitory (*i*)) and two thalamic populations (relay (*s*) and reticular (*r*)). The membrane potential of a population fluctuates $V_{a}(t)$ as a result of the incoming firing rate $\phi_{a}(t)$ from other populations and/or itself according to [14,41,55]

$$\left(\frac{1}{\alpha \beta }\frac{d^{2}}{dt^{2}}+\left(\frac{1}{\alpha }+\frac{1}{\beta }\right)\frac{d}{dt}+1\right)V_{a}(t)=\sum_{a^{\prime }}\nu_{aa^{\prime }}\phi_{a^{\prime }}(t)+\sum_{b}\nu_{ab}\phi_{b}(t-t_{0}/2).$$ (1)

The effect of the presynaptic input on the postsynaptic membrane potential depends on the mean number of synapses between the presynaptic *b* and postsynaptic population *a* (modelled as synaptic strength $\nu_{ab}$) and on the closing and opening rate of synaptic channels characterized by the synaptic decay and rise constants (*α* and *β*). The average postsynaptic membrane potential is transformed at the cell bodies in a population, giving rise to the firing activity *Q*_*a*_(*t*)

$$Q_{a}(t)=\frac{Q_{max}}{1+exp\left(-\left(V_{a}(t)-\theta \right)/\sigma \right)}.$$ (2)

Here, *Q*_*max*_ refers to the maximum firing rate in *Hz*, *θ* is the mean firing threshold in *mV*, and *σ* is the standard deviation of this threshold. This process results in activity propagation in a closed loop between the thalamic and cortical populations, where the firing rate propagated between the thalamus and cortex is delayed by *t*_0_. The firing activity *Q*_*a*_(*t*) was temporally damped using the following expression

$$\left(\frac{1}{\gamma_{a}^{2}}\frac{d^{2}}{dt^{2}}+\frac{1}{\gamma_{a}}\frac{d}{dt}+1\right)\phi_{a}(t)=Q_{a}(t),$$ (3)

with $\gamma_{a}$ being the temporal damping rate, based on $\gamma =v_{a}/r_{a}$, where $v_{a}$ is the propagation velocity and *r*_*a*_ is the mean range of axons. For inhibitory, relay and reticular populations, $\gamma_{a}\approx \infty$, hence $\phi_{a}(t)=Q_{a}(t)$.

### Estimation of model parameters

#### Model power spectrum.

Parameter estimation for nonlinear models remains challenging. Therefore, we transformed the nonlinear model into a linear model by linearizing it around a stable fixed point. Linearization is achieved by expressing the sigmoid function (Equation 2) that transforms $V_{a}(t)$ into *Q*_*a*_(*t*) as Taylor expansion and retaining only the term containing the first derivative ($\rho_{a}$) evaluated at the fixed point. The corresponding details have been discussed previously [14,56]. Using the derivative ($\rho_{a}$), we can express the synaptic strengths as gain parameters in the linear regime $G_{ab}=\rho_{a}\nu_{ab}$, from which we can define loop parameters such as $G_{aba}=G_{ab}G_{ba}$. Accordingly, we transformed the linear system in the time domain to the frequency domain (withk and *ω* being the wave vector and angular frequency) and derived the following transfer function with the firing rate of the excitatory population as output $\phi_{e}(k,\omega )$ and external signal$\phi_{n}(k,\omega )$ as input

$$\frac{\phi_{e}(k,\omega )}{\phi_{n}(k,\omega )}=\frac{G_{es}G_{sn}L^{2}\text{exp}(i\omega t_{0}/2)}{\left(1-G_{srs}L^{2})(1-G_{ei}L)(k^{2}r_{e}^{2}+q^{2}r_{e}^{2}\right)}$$ (4)

Here, *L* follows from the transformation of the second-order differential operator describing the synaptic response (Equation 1) in the Fourier domain, which can be interpreted as a low-pass filter depending on the synaptic parameters *α* and *β*

$$L(\omega )=\frac{1}{\left(1-i\omega /\alpha \right)}\frac{1}{\left(1-i\omega /\beta \right)}$$ (5)

The EEG power spectrum can then be obtained by integrating over k, where the cortex is approximated as a rectangular sheet due to its finite size, with a size of 0.5 m [23]. Using periodic boundary conditions, we derived the following power spectrum [55]

$$P(\omega )=\sum_{m=-\infty }^{m=\infty }\sum_{n=-\infty }^{n=\infty }|\phi_{e}(k_{x},k_{y},\omega ){|}^{2}F(k)\Delta k_{x}\Delta k_{y},$$ (6)

with $k_{x}=\frac{2\pi m}{L_{x}},k_{y}=\frac{2\pi m}{L_{y}}$ and $k=\sqrt{k_{x}^{2}+k_{y}^{2}}$. The filter function *F*(*k*) accounts for the low-pass filtering resulting from volume conduction by the skull, cerebrospinal fluid, and scalp [57]

$$F(k)=e^{-k^{2}/k_{0}^{2}}.$$ (7)

Here, *k*_0_ corresponds to the low-pass cutoff at $k_{0}=10m^{-1}$. This value was obtained in a previous study by using a spherical harmonic head transfer function [57] Lastly, pericranial muscles activity resulted in electromyogram (EMG) artifacts in the EEG, necessitating our attention and correction; hence, $P_{total}(\omega )=P(\omega )$  +  $P_{EMG}(\omega )$. Here, $P_{EMG}(\omega )$ is constrained by an amplitude *A*_*EMG*_ and frequency *f*_*EMG*_ parameter and modelled as

$$P_{EMG}(\omega )=A_{EMG}\frac{(\omega /2\pi f_{EMG}{)}^{2}}{[1+(\omega /2\pi f_{EMG}{)}^{2}{]}^{2}}.$$ (8)

#### Fitting model parameters.

Similarly to [14], a selection of parameters was fixed to reduce parameter space, $Q_{max}=340s^{-1},\gamma_{e}=116s^{-1},\theta =12.9mV,\sigma =3.8mV$. The parameter set $x=[G_{ei},G_{ee},G_{ese},G_{esre},G_{srs},\alpha ,\beta ,t_{0},A_{EMG},f_{EMG}]$ is estimated from EEG data by minimizing the error between the experimentally obtained power spectrum *P*_*exp*_ and model power spectrum *P*_*total*_(*x*) expressed as

$$\chi^{2}=\sum_{j}W_{j}|\frac{P_{total}(x)-P_{exp}}{P_{exp}}{|}^{2},$$ (9)

where *j* denotes the frequency bins. The weights *W*_*j*_ ensure equal weighting for every frequency decade and are proportional to 1/*f*. Because the parameter space was very large, we restricted the parameter values to neurophysiologically plausible values (see reference [14] for values). The $\chi^{2}$ statistic is further transformed into a likelihood function as follows

$$L(x)=\text{exp}[\frac{-\chi^{2}(x)}{2}].$$ (10)

Hence, minimizing the error translates into maximizing the likelihood function. The Markov Chain Monte Carlo algorithm generated a probability distribution for each parameter using Markov Chain random walk [58]. The details of the algorithm have been reported previously [14]. For every subject, we ran the Markov Chain Monte Carlo algorithm to obtain model parameters for individual power spectra. The random walk was initialized using parameters obtained from a large database of healthy controls [59]. This initialization generally does not affect the final output but affects the convergence time. For every subsequent step in the random walk, the likelihood of this step was computed using Equation 9. A new randomly proposed set was generated. The likelihood of this new set of parameters was computed using Equation 9. If these new parameters have a higher probability, this step is used to sample the probability distribution. Otherwise, a random number is drawn from a uniform distribution. If this random number is smaller than the ratio of the probability of the new parameters to that of the old parameters, the probability distribution sampling step is accepted. If this random number is larger than the ratio of the probability of the new parameters to that of the old parameters, the step is not accepted. This procedure was repeated several times until there were no iterative changes in the sampled probability distribution. The test-retest reliability of our estimated parameters is shown in Fig E in S1 File.

### Modelling synaptic plasticity

After estimating the subject-specific mean-field parameters, we performed simulations using a fully nonlinear model (Equations 1–3) to analyze potential routes for neurophysiological recovery in individual patients. In order to do this, we first transformed the subject-specific and estimated gain parameters *G*_*ab*_ to synaptic strength parameters $\nu_{ab}$ using the derivative of the sigmoid function $\rho_{a}$ evaluated at the steady state $G_{ab}=\rho_{a}\nu_{ab}$. The steady-state firing rate for each subject was obtained during the parameter estimation.

We initially explored two possible synaptic plasticity rules based on Hebbian and homeostatic plasticities [15]. Hebbian plasticity refers to a positive-feedback-mediated form of plasticity in which synapses between presynaptic and postsynaptic neurons that are coincidently active are strengthened. Homeostatic plasticity refers to a negative-feedback-mediated form of plasticity, also known as synaptic scaling, which maintains the network activity at a desired set point. Both types of plasticity are thought to occur in neural systems [60]. We first evaluated the Hebbian plasticity using an existing implementation of our current mean-field model, also known as spike-timing-dependent plasticity [17] In addition, we implemented a general homeostatic plasticity rule, as used in previous studies [16,61]

$$\tau_{isp}\frac{d\nu_{ab}}{dt}=-\phi_{a}(t)(\phi_{b}(t)-\rho )$$ (11)

This Equation models the change in synaptic strength $\nu_{ab}$ between populations *a* and *b* as a function of their firing rate and a desired set point *ρ*, which was set to the average firing rate from healthy control subjects that were included for this study. The synaptic time-scale parameter $\tau_{isp}$ was chosen to match previous literature and was set to $\tau_{isp}=20$ [16]. Simulations of Equations 1–3 were first run for 30 s without plasticity, followed by plasticity for the remaining 10 min. First, we examined spike-timing-dependent plasticity. However, spike-timing-dependent plasticity in isolation leads to an unopposed strengthening of synapses, resulting in an unstable system. Next, we executed both spike-timing-dependent plasticity and homeostatic plasticity but noticed that stability only occurred if the contribution of homeostatic plasticity was very large and that of spike-timing-dependent plasticity was negligible. Hence, in the results presented in our manuscript, we reported only homeostatic plasticity. Simulations of the model are performed by solving Equations 1–3 and 11 using an Euler(-Maruyama) solver with a sufficiently small time step (1×10-4) using in-house Matlab scripts (R2021a). The Laplace operator in Equation 3 is set to zero to run the model as a neural mass model instead of a neural field model.

### Statistics

We test for significant differences for parameters $(G_{ei},G_{ee},G_{ese},G_{esre},G_{srs},\alpha ,\beta ,$ and *t*_0_) between groups using the Wilcoxon rank-sum test. The false discovery rate was subsequently used to correct for multiple tests [62]. We quantified the similarity between the power spectra for different groups using the Kullback-Leibler divergence [63]. Summary statistics from the power spectra, such as the aperiodic exponent, peak frequency, and peak power, were extracted using the FOOOF algorithm [5]. We used the following settings for the FOOOF algorithm (maximum number of peaks = 2, minimum peak height 0.3 (units of power), peak width limits 1-14 (Hz), aperiodic mode = “knee”). We tested the null hypothesis that the presence of a spectral peak was equal between MCS and UWS using the chi-square test. Finally, we tested the relationships between these summary statistics and the metabolic indices using Pearson’s correlation coefficients.

### Machine learning classification

We utilized a random forest classifier to analyze which parameters most contribute to the diagnosis of MCS versus UWS in a multivariate manner. The random forest classifier is a supervised method that uses multiple (bootstrap-aggregated or bagged) decision trees to solve a classification problem [64]. Classification is based on the majority vote of the decision trees, which helps minimize overfitting. Out-of-bag error was used to determine the optimal number of trees, which was set to 40. The predictive value was evaluated using 5-fold cross-validation, in which 80% of the data were used as a training set and 20% of the data as a test set. Classification results were evaluated using the area under the curve (AUC) of the receiver operator characteristic (ROC) curve and using specificity and sensitivity. Results for only the test set are reported. Feature selection was subsequently performed using a backward elimination approach: a prediction model was trained using all features, and for every subsequent step, the feature with the lowest importance from the previous step was eliminated, and the prediction model was retrained. This allowed us to keep track of the AUC as a function of the number of features eliminated. A substantial drop in the AUC (based on visual inspection) was used as a stop criterion, allowing us to determine the eventual number of features. To determine if the obtained AUC value exceeded chance levels, we generated surrogate AUC values by reclassifying the data and randomly permuting group memberships (MCS vs. UWS). This process created a null distribution of 10,000 AUC values, which we used to test the validity of the genuine AUC value.

### Acknowledgments

We thank the patients and their families who agreed to participate in this study. The authors also thank the staff of the Nuclear Medicine Department at the University Hospital of Liege, especially Roland Hustinx and Claire Bernard. We are highly grateful to the members of the Liège Coma Science Group/Centre du Cerveau for their assistance in the clinical evaluations, especially Cecile Carette, and the clinicians from the Intensive Care Unit of the University Hospital of Liege, especially Didier Ledoux, Paul Massion, and Gaelle Tronconi.

## Supporting information

S1 File This supplementary file provides detailed examples and validation analyses of subject-specific model fitting and parameter estimation in patients with disorders of consciousness (DoC). Figs A–C illustrate the MCMC-derived parameter distributions and power spectrum fits for representative subjects across clinical categories (UWS, MCS, EMCS), highlighting characteristic spectral peaks and parameter selection in corticothalamic loop components. Fig D summarizes estimated model parameters across etiologies, while Fig E evaluates the robustness, reproducibility, and specificity of parameter estimation. Finally, Fig F presents correlations between model parameters and metabolic indices, assessing potential physiological associations.
(PDF)
