# Supplementary material for: Individual trajectories for recovery of neocortical activity in disorders of consciousness
Source: PLoS Comput Biol. 2025 Nov 11;21(11):e1013659. doi: 10.1371/journal.pcbi.1013659 (PMC12622836; doi:10.1371/journal.pcbi.1013659)
Supplement: S1 File — Figs A–C illustrate the MCMC-derived parameter distributions and power spectrum fits for representative subjects across clinical categories (UWS, MCS, EMCS), highlighting characteristic spectral peaks and parameter selection in corticothalamic loop components. Fig D summarizes estimated model parameters across etiologies, while Fig E evaluates the robustness, reproducibility, and specificity of parameter estimation. Finally, Fig F presents correlations between model parameters and metabolic indices, assessing potential physiological associations. (PDF) [file pcbi.1013659.s001.pdf]

Supplementary material: Individual trajectories for recovery of neocortical activity in disorders of consciousness

Prejaas K.B. Tewarie, Romesh Abeysuriya, Rajanikant Panda, Pablo Nùñez, Marie M. Vitello, Glenn van der Lande, Olivia Gosseries, Aurore Thibaut, Steven Laureys, Gustavo Deco, Jitka Annen

Example fits for individual subjects

Parameter estimates and estimates of the corresponding power spectra for a few subjects is shown below. The Markov Chain random walk estimates the probability distribution for parameters and their joint probability distribution. Figs A-C below show the probability distribution of the different parameters, together with some aggregate measures X, Y, Z, which correspond to the intracortical loop, corticothalamic loop and intrathalamic loop. These measures are defined in terms of the synaptic gains

$$X = \frac{G_{ee}}{1 - G_{ei}}, \quad Y = \frac{G_{ese} + G_{esre}}{(1 - G_{srs})(1 - G_{ei})}, \quad Z = -G_{srs}\frac{\alpha\beta}{(\alpha + \beta)^2}.$$

The blue curves in Figs A-C correspond to the estimated probability distributions for the individual, whereas the red line reflects the optimal value in accordance with the joint probability distribution. Note that the estimated spectra in the left lower panel are smooth (red curve), while the empirical power spectra (blue curves) contain a noise floor. Hence, our parameter estimation method captures the global pattern of the power spectra and the most prominent spectral peaks. While this could lead to omission of details in the estimated power spectra, this also avoids overfitting. From most estimated spectra, we can observe that there is an underestimation of upper beta and gamma activity. An important contribution could be muscle artefacts.

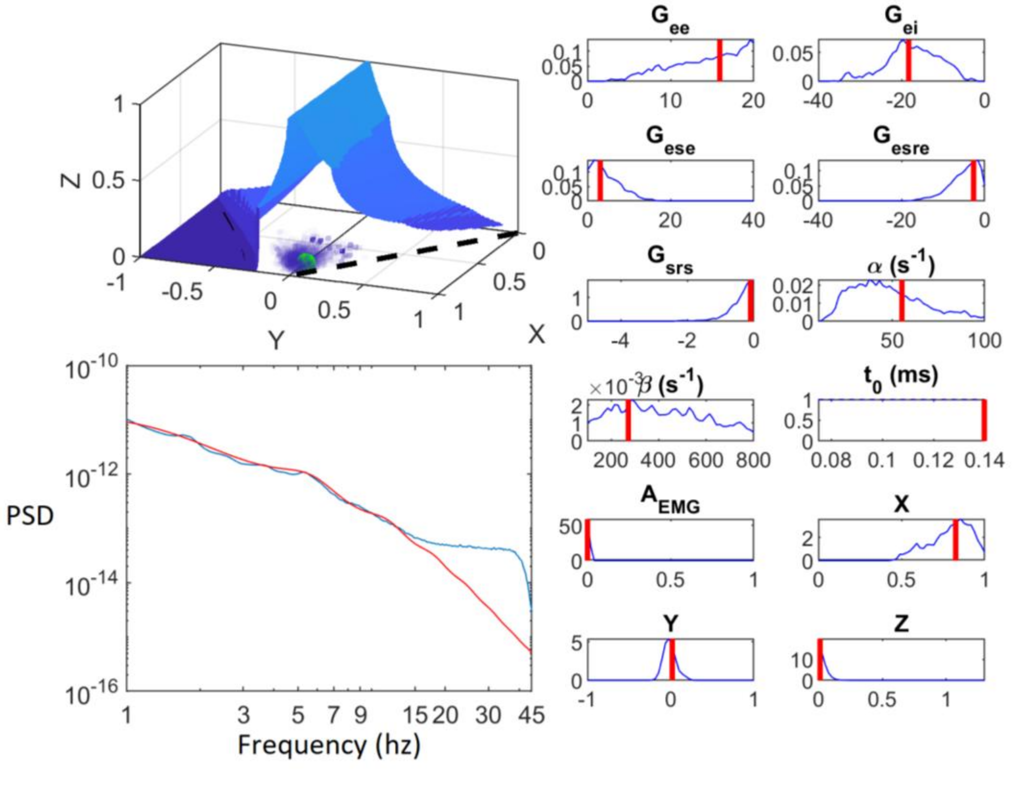

Fig A. Example of a fit for an individual subject with UWS. *The right panel shows the individual probability distribution of the parameters together with the (selected parameter) based on the joint probability distribution. The upper left panel shows the values of the distributions X, Y, Z in the parameter space together with the selected set of parameters (green dot). X, Y, Z refer to the intracortical loop, corticothalamic loop and intrathalamic loop.*

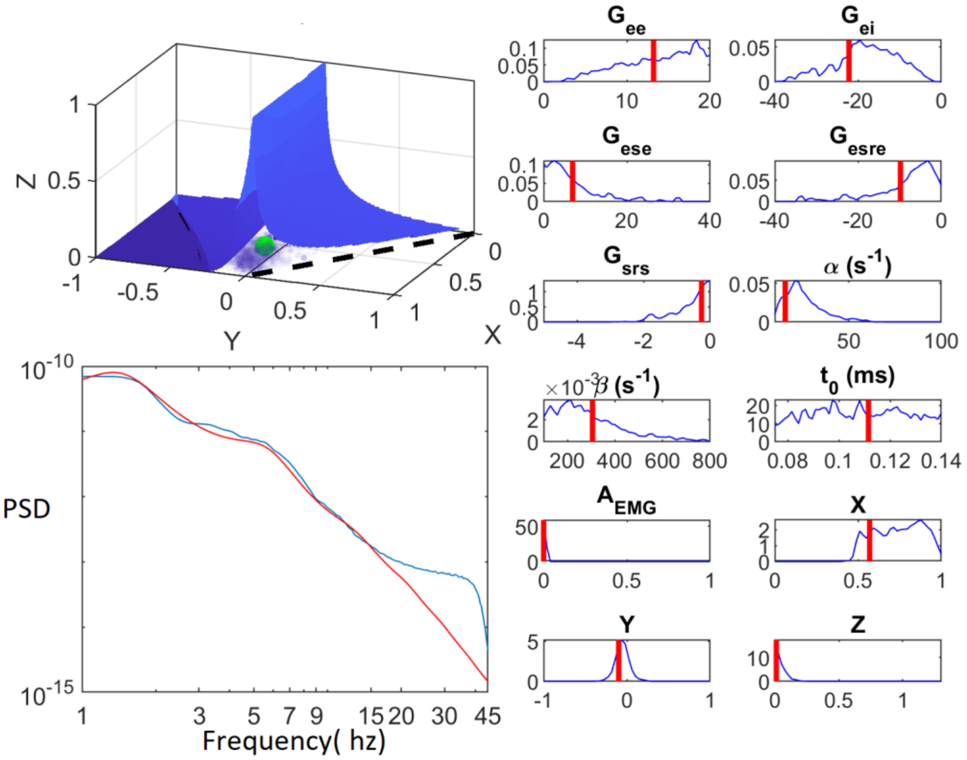

Fig B. Example of a fit for an individual subject with MCS. *The right panel shows the individual probability distribution of the parameters together with the (selected parameter) based on the joint probability distribution. The upper left panel shows the values of the distributions X, Y, Z in the parameter space together with the selected set of parameters (green dot). X, Y, Z refer to the intracortical loop, corticothalamic loop and intrathalamic loop. A clear theta peak is observed for both the empirical and estimated power spectrum.*

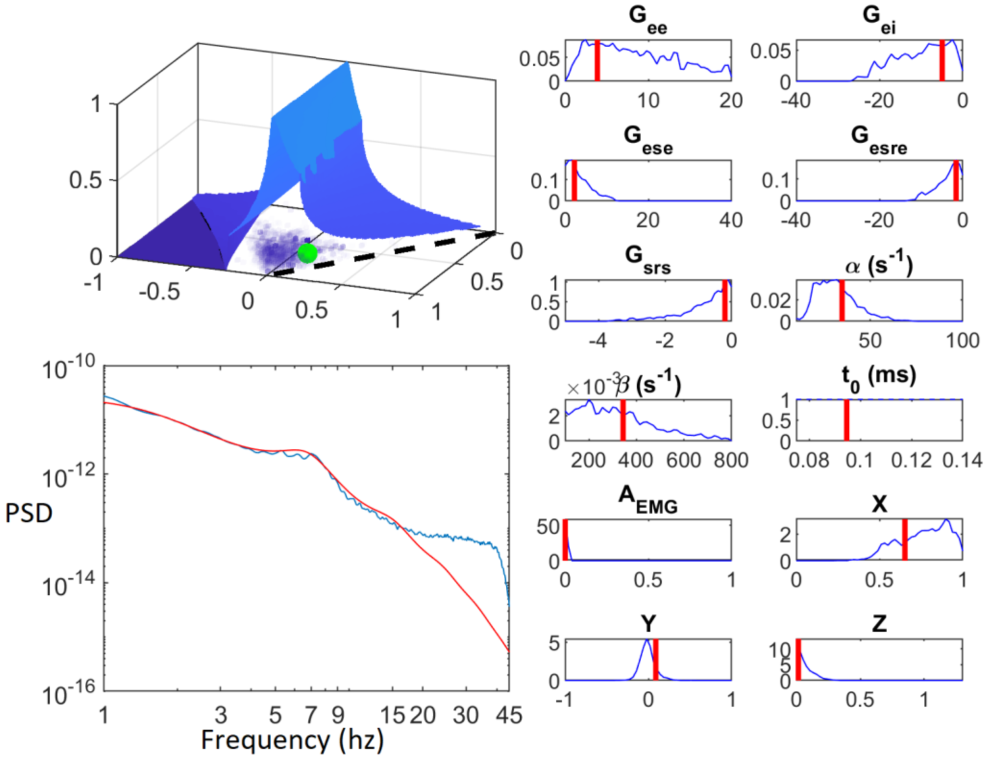

Fig C. Example of a fit for an individual subject with EMCS. *The right panel shows the individual probability distribution of the parameters together with the (selected parameter) based on the joint probability distribution. The upper left panel shows the values of the distributions X, Y, Z in the parameter space together with the selected set of parameters (green dot). X, Y, Z refer to the intracortical loop, corticothalamic loop and intrathalamic loop. A clear theta/alpha peak is observed for both the empirical and estimated power spectrum.*

Estimating corticothalamic model parameters for disturbed EEG patterns in patients with DoC for different etiologies

We estimated power spectra for every subject (averaged across channels) using parameter estimation of the corticothalamic biophysical model. Fig D shows the results of all parameters for three different etiology groups: traumatic brain injury, anoxia after cardiac arrest and all remaining etiologies grouped together. Note that we consider exactly the same estimated parameters as depicted in Fig 2, but now grouped according to etiology. Note that no clear difference was found for any of the corticothalamic parameters (G_{ESE} and G_{ESRE}) as was seen for the DoC groups.

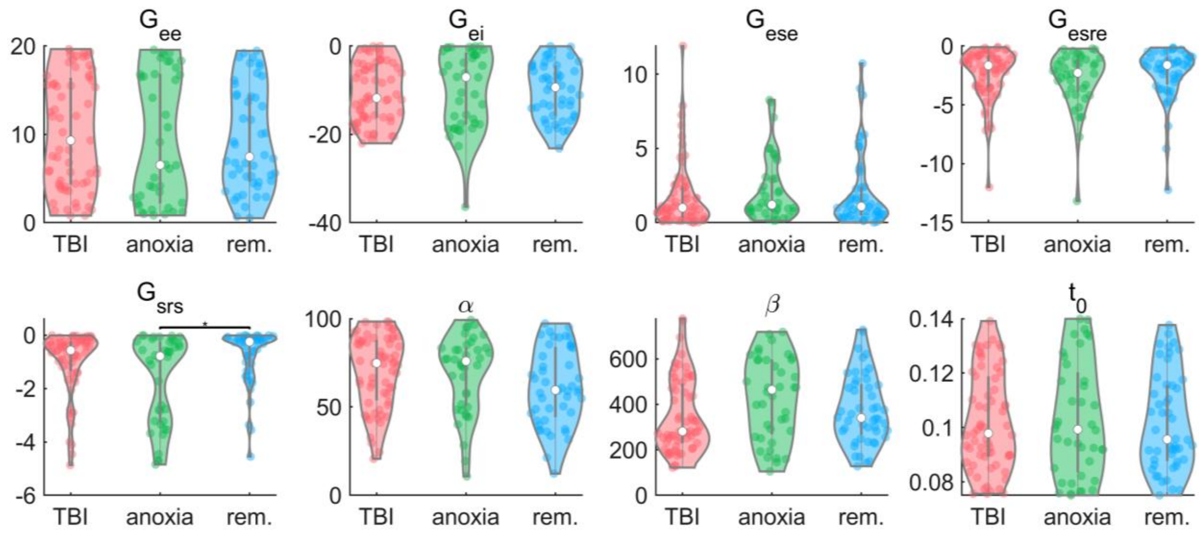

Fig D. Estimating corticothalamic model parameters in DoC for different etiologies. *The panels show the parameter estimates in the different groups. Blue circles in the UWS group denote MCS* patients. Abbreviations: traumatic brain injury (TBI), remaining etiologies (rem.), excitatory corticothalamic synaptic strengths (G_{ESE}), inhibitory corticothalamic synaptic strengths (G_{ESRE}), Excitatory cortical synaptic strengths (G_{EE}), inhibitory cortical synaptic strengths (G_{EI}), intrathalamic synaptic strengths (G_{SRS}), synaptic decay and rise constants (α and β), corticothalamic time delay t_0.*

Test-retest reliability of estimated parameters

We ran the MCMC algorithm twice to test the robustness of the goodness-of-fit and estimated parameters. The left panel in Fig E shows the error defined as the relative difference between model parameters from run one and rune two. This is define as the absolute value of ((Parameter run 1) - (Parameter run 2)/(Parameter run 2) * 100). The left panel shows that the error in the estimate is small, and for most parameters the mean does not exceed 0.5%. The slight difference in parameter estimates did not change goodness-of-fit χ (Mann-Whitney-U test U =0.1, $p > 0.05$). In order to test whether parameter estimates are associated with local minima we fitted a unimodal and bimodal distribution to every parameter distribution. The middle panel in Fig E shows the Akaike Information Criterion for the bimodal and unimodal fits. The fit for the bimodal distribution did not outperform the fit for the unimodal distribution (Mann-Whitney-U test U =0.05, $p > 0.05$). Hence, this suggests that the obtained parameter values may correspond to global minima rather than local minima.

Lastly, we evaluated the model performance for a subject based on parameters from a different subject. The right panel of Fig E shows that model parameters from a different subject are far worse in estimation of a subject's power spectrum than parameters from the same subject (Mann-Whitney-U test U = 4, $p < 0.001$). Hence, from this we can conclude that the estimated parameters are subject specific.

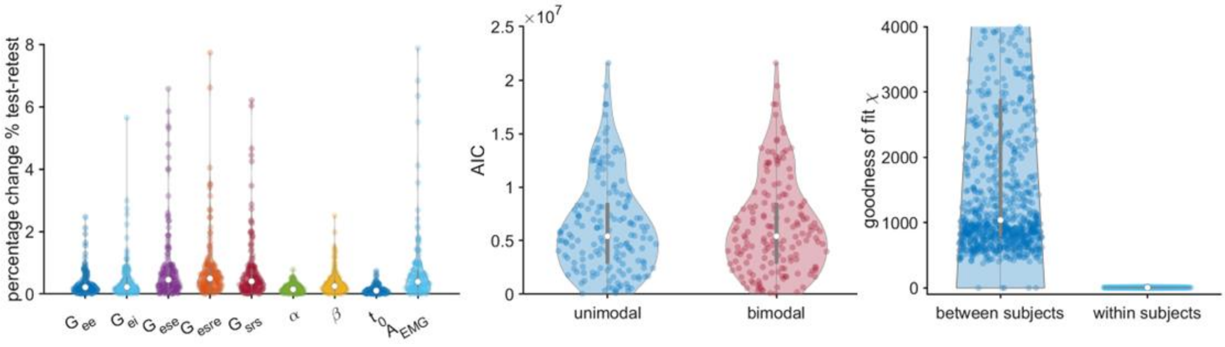

Fig E: Robustness and Specificity of MCMC-Derived Parameter Estimates. *The left panel illustrates the error in parameter estimates from two separate runs of the MCMC algorithm, defined as the relative difference. Errors are minimal, with mean differences not exceeding 0.5%, indicating stable parameter estimation across runs (Mann-Whitney-U test U = 0.1, p > 0.05). The middle panel compares the Akaike Information Criterion (AIC) for unimodal versus bimodal fits of parameter distributions, showing no significant difference in fit quality (Mann-Whitney-U test U = 0.05, p > 0.05), suggesting the parameters likely represent global minima. The right panel assesses model performance using parameters from different subjects, demonstrating significant deterioration in fit (Mann-Whitney-U test U = 4, p < 0.001), thus confirming the subject-specific nature of the estimated parameters.*

Associations between mean-field model parameters and metabolic integrity

We examined each fitted parameter against the PET-derived metabolic index. We used the same parameter set G_{ee}, G_{ei}, G_{ese}, G_{esre}, G_{srs}, α, β, t_0, EMG_a. Results indicate that most Spearman coefficients are near zero, except for the excitation in the corticothalamic loop G_{ESE}.

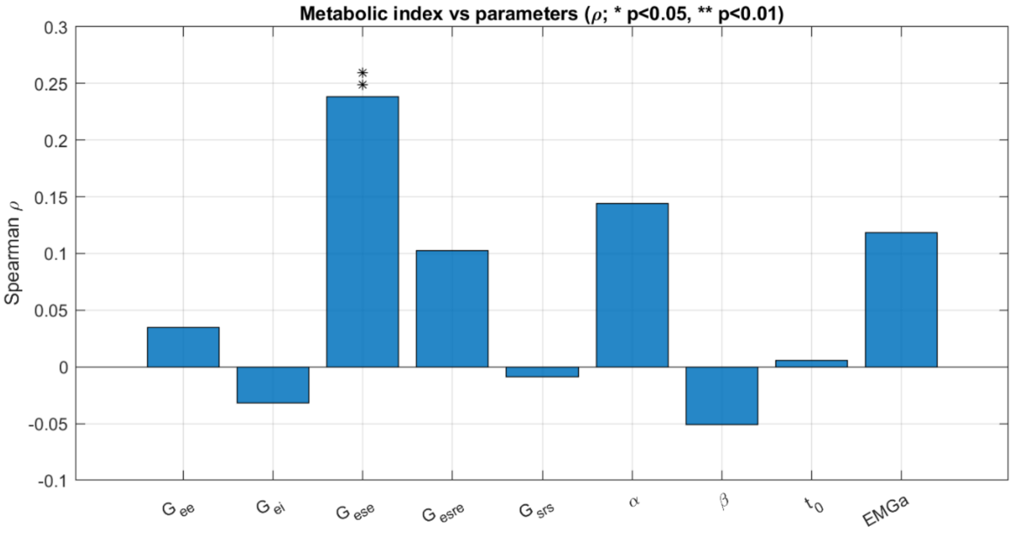

Fig F: Summary of monotonic associations between mean-field model parameters and metabolic index. *Bars show Spearman correlation coefficients (ρ) for each parameter. Asterisks denote significance thresholds (* $p<0.05$; ** $p<0.01$). Most parameters cluster near $\rho \approx 0$, indicating absence of clear associations at the group level, while only the excitatory gain in the corticothalamic loop G_{ESE} shows a significant correlation.*
